# Studying the electronic and phononic structure of penta-graphane

**DOI:** 10.1080/14686996.2016.1219970

**Published:** 2016-10-07

**Authors:** Hamideh Einollahzadeh, Seyed Mahdi Fazeli, Reza Sabet Dariani

**Affiliations:** ^a^ Department of Physics, Alzahra University, Tehran, Iran; ^b^ Department of Physics, University of Qom, Qom, Iran

**Keywords:** Penta-graphane, density functional theory, G_0_W_0_ approximation, band gap, phonon structure, specific heat capacity, 60 New topics/Others, 104 Carbon and related materials

## Abstract

In this paper, we theoretically consider a two dimensional nanomaterial which is a form of hydrogenated penta-graphene; we call it penta-graphane. This structure is obtained by adding hydrogen atoms to the sp^2^ bonded carbon atoms of penta-graphene. We investigate the thermodynamic and mechanical stability of penta-graphane. We also study the electronic and phononic structure of penta-graphane. Firstly, we use density functional theory with the revised Perdew–Burke–Ernzerhof approximation to compute the band structure. Then one–shot GW (G_0_W_0_) approach for estimating accurate band gap is applied. The indirect band gap of penta-graphane is 5.78 eV, which is close to the band gap of diamond. Therefore, this new structure is a good electrical insulator. We also investigate the structural stability of penta-graphane by computing the phonon structure. Finally, we calculate its specific heat capacity from the phonon density of states. Penta-graphane has a high specific heat capacity, and can potentially be used for storing and transferring energy.

## Graphical abstract

**Figure:**
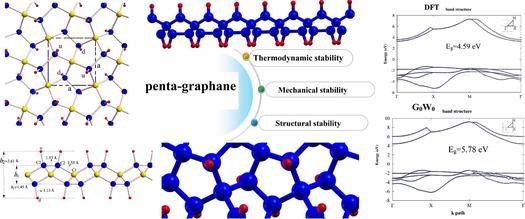

## 1. Introduction

Carbon is interesting for scientists because of its benign environmental properties and its abundance in the nature. Carbon can bond with itself in at least three different ways, which leads to three different allotropes. In diamond each carbon atom is strongly bonded to the four adjacent atoms via sp^3^ hybridization; in graphite each carbon atom is bonded to its three neighbors via sp^2^ hybridization.[1,2] *π* bonds are perpendicular to plane and sp^2^ bonds are in plane. In carbon nanotube, curvature induces rehybridization, so we have no longer pure *π* bonds. Therefore, higher curvature leads to more rehybridization. As stated in [3], ’the degree of sp^3^-sp^2^ rehybridization depends on the degree of curvature’ (p. 244).

In 2004, graphene, a single-atom-thick layer of carbon, was isolated from bulk graphite by scientists at Manchester University, with a very simple and effective method, namely the ‘scotch tape method’.[4]

Graphene is a 2D crystal of carbon. In graphene, carbon atoms are densely packed into a two-dimensional honeycomb crystal lattice.[5] It is a semimetal, with zero band gap [6] and is considered to be a super-material for its unique properties. Graphene is the thinnest, lightest and strongest material ever discovered.[7] It is transparent [8] and conducts electricity much better than copper.[9] These unique properties have attracted many researchers. One of the interesting areas in these studies is investigating new graphene allotropes, since 2D dimension nanostructures are ideal for modern electronics.[10–13]

In 2012, a new phase of carbon was reported.[14] T12-carbon is a tetragonal allotrope of carbon, with 12 atoms in unit cell, this structure uses sp^3^ bonding and has spread helical six-membered rings interconnected by pair of five and seven-membered rings. T12-carbon has not been synthesized so far, although in [14] the authors showed that it is stable under certain conditions and has the physical properties of diamond phase. In early 2015, penta-graphene, a 2D graphene allotrope with a pentagon structure, was proposed by an international group of researchers.[15] They suggested that penta-graphene may be exfoliated from T12-carbon. The band gap of penta-graphene was calculated as 4.1–4.3 eV using the G_0_W_0_ approximation.[16] Penta-graphene is meta-stable and has unique properties, making it an interesting topic to study; however, it has not yet been synthesized. Recently, in [17], the authors have shown that penta-graphene cannot be made experimentally, and it rapidly restructures toward graphene. In this article, we study the properties of hydrogenated penta-graphene.

Penta-graphene has sp^2^ and sp^3^ bonded carbon atoms. The sp^2^ bonded carbons have got an extra electron, so we can hydrogenate penta-graphene. The fully hydrogenated graphene was called graphane;[18,19] therefore, we call this structure penta-graphane. Here, we study the thermodynamic and mechanical stability of penta-graphane and we use density functional theory (DFT) and G_0_W_0_ approximation for determining the electronic structure and properties of penta-graphane.

Also, we study the phonon dispersion and the phonon density of states for penta-graphane using DFT-GGA (RPBE) calculation in the framework of the pseudopotential approach. Then we compare the heat capacity of penta-graphene and penta-graphane.

## 2. Computational method

All calculations in this work are carried out with the *ab initio* ABINIT package.[20] The ABINIT wave functions are expanded in plane waves. The Brillouin zone sampling k point mesh parameter is based on the Monkhorst-pack grid.[21] We do a convergence test with respect to the energy cutoff and k-point mesh. We have a two-dimensional unit cell, since we have to use the supercell technique in ABINIT.

DFT is a valuable method for describing many-body systems through the electron density. In Kohn–Sham-DFT (KS-DFT), an interacting many electron system is reduced to a model of non-interacting electron system. Many-body effects such as Coulomb interaction, Hartree energy and exchange correlation energy are included in the effective potential. The generalized gradient approximation (GGA) is an approach for estimating exchange correlation (xc). In GGA, exchange correlation energy depends on both the electron density and its gradient. The DFT is based on the ground electronic state of a system; therefore DFT often underestimates the band gap of semiconductors and insulators.[22,23]

GW is a good method for correcting the band structure. This method relies on a perturbative treatment starting from the DFT. In GW, the self-energy is approximated as the product of Green function G and the screened Coulomb interaction W within the random phase approximation.[24] The self-consistent GW computations are cumbersome, so the most commonly approximate is applying a first-order perturbation approach called one-shot GW (G_0_W_0_). In the G_0_W_0_, the quasiparticle wave functions can be approximated by the Kohn–Sham orbital, thus the quasiparticle energy becomes:

(1) $$E_{i}=\varepsilon_{i}+Z_{i}\langle \Phi_{\text{i}}\left|\Sigma \left(\varepsilon_{i}\right)-V_{xc}\right|\Phi_{\text{i}}\rangle$$

where $\Sigma =G_{0}W_{0}$ and Z_i_ is the renormalization factor.[25,26]

The phonon frequency ω, as a function of the phonon wave vector **q**, is the solution of the following secular equation for the force constant $C_{s\left(\alpha \right),t\left(\beta \right)}$ :

(2) $$det\left|\frac{1}{\sqrt{M_{s}M_{t}}}C_{s\left(\alpha \right),t\left(\beta \right)}\left(q\right)-\omega^{2}\left(q\right)\right|=0$$

Here, *M*_*t*_ and *M*_*s*_ are the atomic masses. The dynamical matrix is denoted by:

(3) $$C_{s\left(\alpha \right),t\left(\beta \right)}\left(q\right)=\frac{\partial^{2}E}{\partial u_{s\left(\alpha \right)}^{}\left(q\right)\partial u_{t\left(\beta \right)}\left(q\right)}$$

where $u_{s\left(t\right)}^{\alpha \left(\beta \right)}$is the displacement of atom *s*(*t*) in $\alpha \left(\beta \right)$ direction. A discrete Fourier transport leads to the real space force constant. We can use the dynamical matrix in real space or reciprocal space for the phonon calculation. Useful details on investigating the response to atomic displacement can be found in [27].

## 3. Results and discussion

**Figure 1.**
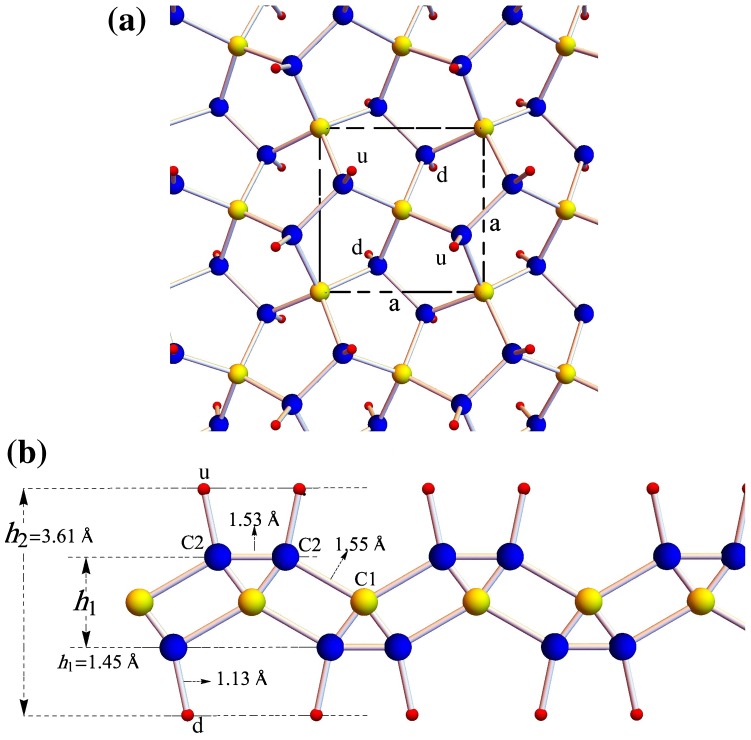
(a) Top view and (b) side view of the atomic structure of penta-graphane. Yellow, blue and red sphere represent C1, C2 and hydrogen atoms, respectively.

By hydrogenating penta-graphene, a new 2D crystalline phase can be obtained, which we call penta-graphane because of its similarity to graphane. As we mentioned, carbon atoms in penta-graphene have two sp^2^ and sp^3^ hybridizations. Hence by adding hydrogen atoms to the sp^2^ bonded carbon atoms, penta-graphane has only sp^3^ hybridization. The symmetry of penta-graphene is P-42_1_ m, which can be specified by tetragonal lattice, thus hydrogenation of penta-graphene does not change the symmetry. The unit cell of penta-graphane consists of six carbons and four hydrogen atoms (the ratio of C/H is 3/2), which are distributed symmetrically in penta-graphane. This structure is depicted in Figure 1(a); the unit cell of the penta-graphane is shown with dashed line. Hydrogen atoms labeled by ‘d’ points are located at the bottom of the C2 atoms, and hydrogen atoms labeled by ‘u’ points are located at the top of the C2 atoms.

We compute the optimum structure of penta-graphane by ABINIT, the optimized lattice constants are found to be a=b=3.60Å, which are 1% smaller than penta-graphene. Penta-graphane has two types of carbon atoms, C1 has sp^3^ bonds with four carbons and C2 has sp^3^ bonds with three carbons and one hydrogen. The bond length of (C1–C2) is 1.55Å, (C2–C2) is 1.53 Å and (C2–H) is 1.13Å. The bond angle $\theta_{C2-C1-C2}$ is 124.2^°^, the thickness of this structure becomes 3.61Å. After hydrogen is added to the sp^2^ carbon atoms in penta-graphene they become sp^3^ hybridized. The out-of-plane movements of carbons increase, which tends to decrease the lattice constants. The C2–C2 double bond becomes a single bond; its length increases, which tends to increase the lattice constants. Finally, in our computations, the optimized lattice constants are decreased by about 1%, in comparison with penta-graphene lattice constants.

**Figure 2.**
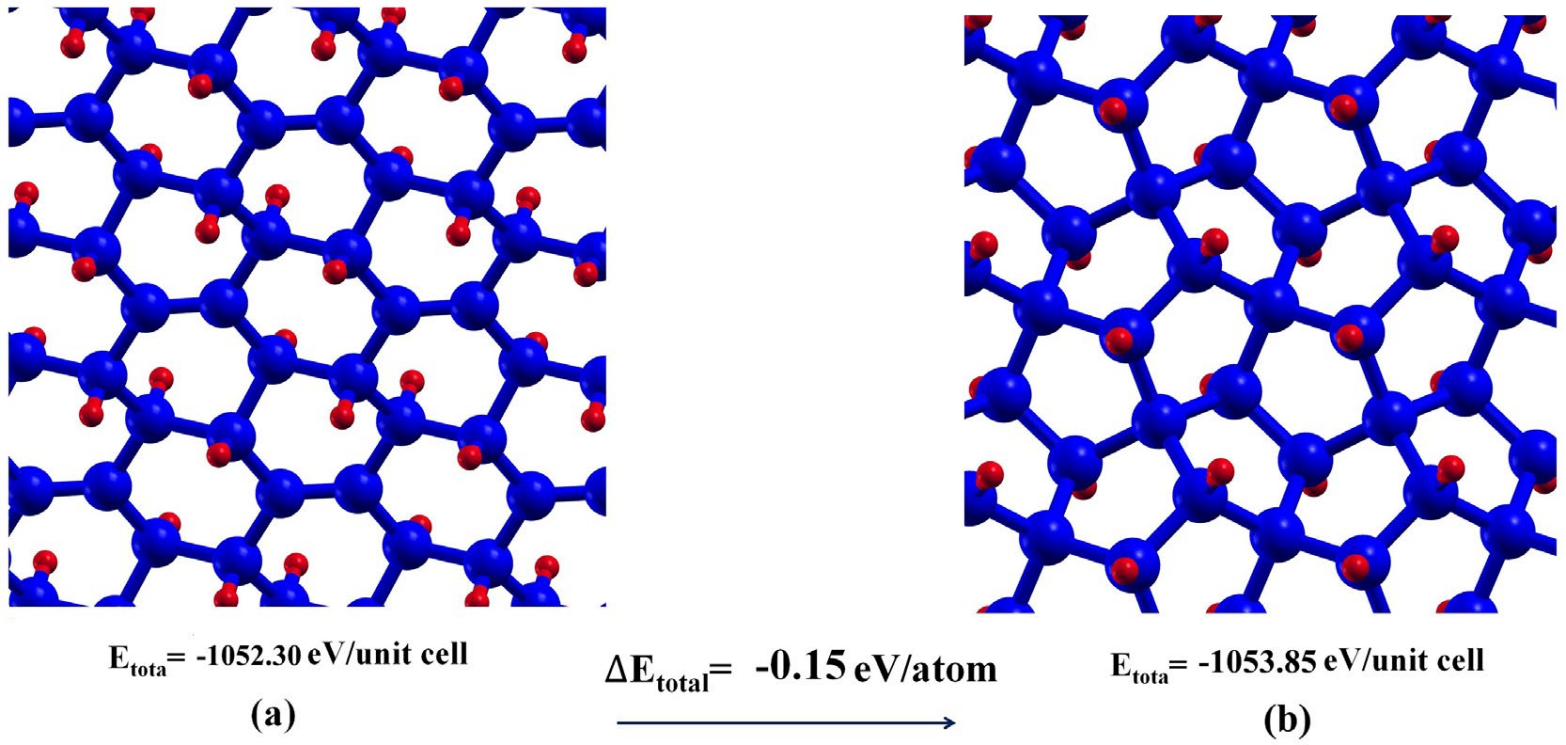
Total energy of (a) C_6_H_4_ (not fully hydrogenated graphane) and (b) penta-graphane. Blue and red spheres represent carbon and hydrogen atoms, respectively.

To consider the thermodynamic stability of penta-graphane, we compare total energy per unit cell of penta-graphane and not fully hydrogenated graphane with six carbon atoms and four hydrogen atoms in Figure 2. According to our KS-DFT calculation, penta-graphene is 0.9 eV/atom less stable than graphene, while penta-graphane is more stable than not fully hydrogenated graphane (C_6_H_4_).

Penta-graphene is meta-stable,[16] but hydrogenation of penta-graphene leads to a more stable structure (penta-graphane). Indeed, we remove free bonds by hydrogenating penta-graphene and sp^2^ bonds change to sp^3^ bonds. C-H formation in penta-graphane decreases energy and removes free bonds. Therefore, penta-graphane has lower energy than penta-graphene. Penta-graphane is 0.15 eV/atom more stable than the not-fully hydrogenated graphane (C_6_H_4_). In penta-graphane, four C-H bonds have got approximately similar angle bonds that lead to more symmetries and more stable structure than C_6_H_4_.

We also study the stability of penta-graphane with respect to other compounds through formation energy, as in [18]. The total energy is computed using DFT-GGA-RPBE and Martins-Troullier pseudopotential, which is defined below. According to our calculations, total energies of isolated carbon and hydrogen are –156.01 eV and –12.63 eV, respectively. We report the binding energy (the difference between the total energy of the isolated atoms and the total energy of the compounds) and formation energy of the compounds from crystalline graphite and gaseous molecular hydrogen in Table 1.

**Table 1.** Comparison of the total energy, binding energy and formation energy calculated by the DFT-GGA-RPBE method between penta-graphane and three other structures with the same stoichiometry.

	**Total energy (eV/unit cell)**	**Binding energy (eV/atom)**	**Formation energy (eV/atom)**	**Formation energy (kJ mol**^**–1**^**)**
**Penta-graphane**	−1053.85	−6.73	+0.03	+28.95
**Not fully hydrogenated graphane (C**_**6**_**H**_**4**_**)**	−1052.30	−6.57	+0.19	+183.3
**p-benzyne**	−1048.99	−6.24	+0.52	+501.7
**p-phenylene**	−1053.42	−6.68	+0.08	+77.19
**graphite**	−660	−8.99	0	0
**H**_**2**_	−32.09	−3.415	0	0

Table 1 indicates that penta-graphane has lower formation energy in comparison with three other structures of the same stoichiometry. Therefore, our results imply that penta-graphane is more stable than three other structures with the same stoichiometry. The p-phenylene structure has synthesized experimentally.[28] The formation energy of penta-graphane is slightly smaller than p-phenylene. Therefore the possibility of synthesizing penta-graphane increases.

We also compare penta-graphane and two other possible candidate forms of hydrogenated graphene in Table 2. Graphane in the chair conformation sheet is more stable than boat conformation sheet. Other candidate forms for hydrogenated graphene have more formation energy than the chair-graphane sheet.[29] Accordingly we compare penta-graphane with the chair-graphane sheet in Table 2. Our result for the formation energy of the chair-graphane sheet is in agreement with [30]. The formation energy of the penta-graphane is similar to the formation energy of the chair-graphane. The small positive formation energy of penta-graphane indicates that, under typical experimental conditions, it might be expected to be synthesized.

**Table 2.** Comparison of the total energy, binding energy and formation energy calculated by the DFT-GGA-RPBE method between penta-graphane and two other possible candidate forms of hydrogenated graphene.

	**Total energy (eV/unit cell)**	**Binding energy (eV/atom)**	**Formation energy (eV/atom)**	**Crystal structure**
**Penta-graphane**	−1053.85	−6.73	+0.03	a=b=3.60 Å c=10 Å
**Not fully hydrogenated graphane (C**_**6**_**H**_**4**_**))**	−1052.30	−6.57	+0.19	a=b=4.22 Å c=10 Å
**Fully hydrogenated graphene (chair-graphane sheet) (C**_**2**_**H**_**2**_**)**	−362.08	−6.2	+0.002	a=b=2.51 Å c=15 Å

A study of the mechanical properties of penta-graphane [15] indicates that penta-graphane satisfies conditions of mechanical stability (*C*_11_ *C*_22_-*C*_12_^2^ > 0, *C*_66_>0) (*C*_11_ = *C*_22_ = 223 GPa nm, *C*_12_ = 55 GPa nm and *C*_66_ = 157 GPa nm). *C*_12_ (the component of the elastic modulus tensor) is positive for penta-graphane. Therefore we have a positive Poisson’s ratio $\left(\frac{C_{12}}{C_{11}}≃0.24.\right)$ The in-plane Young’s modulus of penta-graphane is 209 GPa nm. The out-of-plane movements increase in penta-graphane, and consequently the in-plane Young’s modulus becomes smaller in penta-graphane than in penta-graphene.

### 3.1. Electronic structure

Here we compute the band structure of penta-graphane using DFT-GGA by the ‘revised Perdew–Burke–Ernzerhof’ type (RPBE) functional [31] (based on the Kohn–Sham eigenvalues). The vacuum along the *z* direction is taken to be 10 Å, which is sufficiently large for convergence the total energy.[32] The plane wave basis set is used with a cutoff energy of 50 Hartree, the total energy of which converges to 1 meV/atom. The Brillouin zone is sampled by a 18 × 18 × 1 k-point mesh.

**Figure 3.**
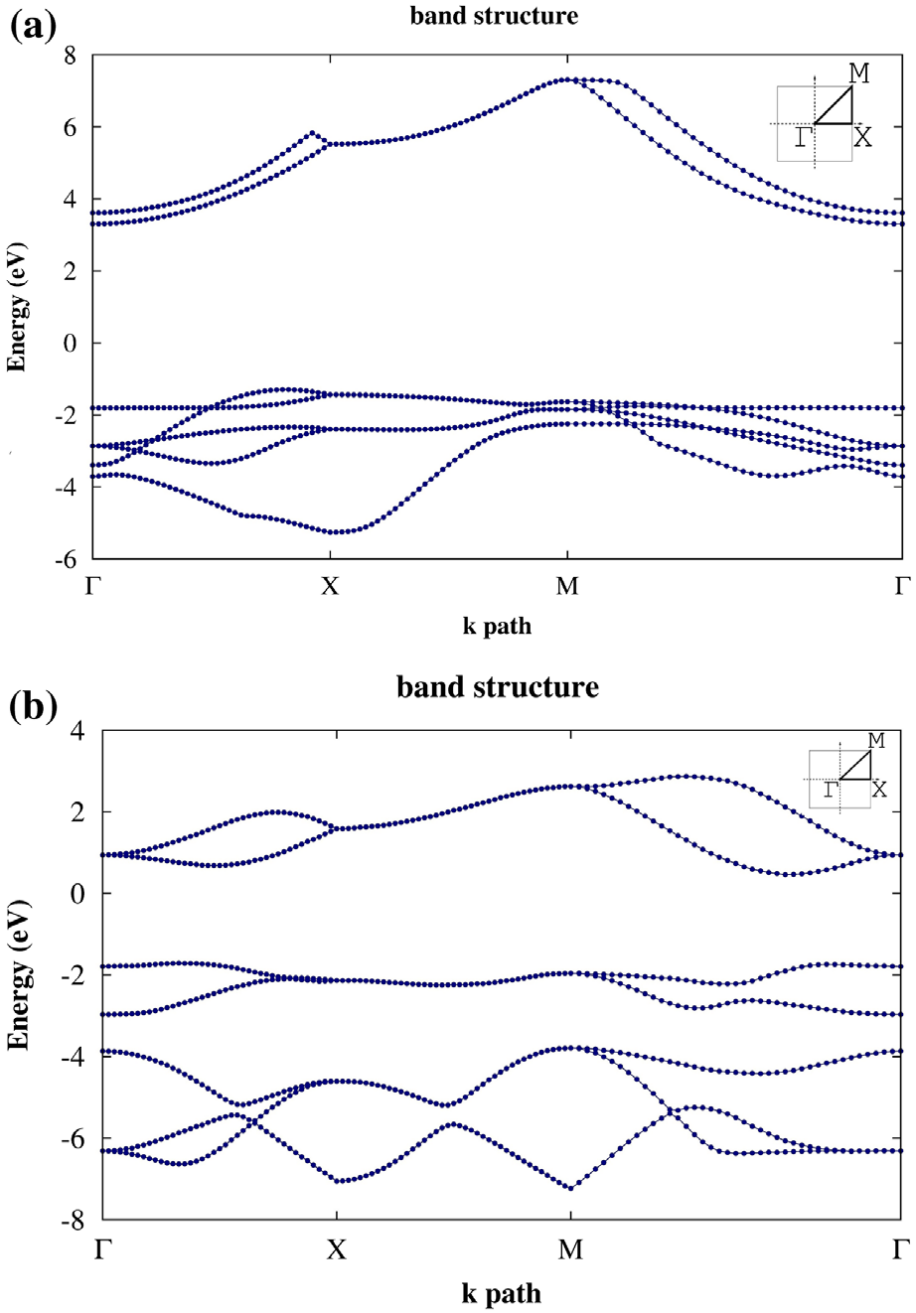
(a) The band structure of penta-graphane and (b) penta-graphene [11] calculated by using DFT-GGA (RPBE) around Fermi energy.

From DFT-GGA (RPBE) calculations we conclude that penta-graphane has an indirect band gap; its valence band maximum (VBM) is (0.389 0 0) close to the X point in Г-X path, and its conduction band minimum (CBM) is in the Г point. Here, we apply Martins–Troullier pseudopotential [33] in which 1s electrons are applied in potential. By considering penta-graphane unit cell, we have 28 electrons, so penta-graphane has 14 filled bands and its band gap is located between 14th and 15th levels. The band gap of penta-graphane becomes 4.59 eV by DFT-GGA (RPBE) approximation. The difference between the indirect and direct gap is 0.5 eV. The band structure of penta-graphane around Fermi level by DFT-GGA (RPBE), along symmetry line Г–X-M-Г (with (Г=(0,0,0)2π/a, X=(1/2,0,0)2π/a, and M=(1/2,1/2,0)2π/a)) is shown in Figure 3(a).

Penta-graphane has the bigger band gap than the penta-graphene. This gap is originated from the full sp^3^ bonding in penta-graphane. Therefore, since all the electrons participate in bonds, all the orbitals are localized.

**Figure 4.**
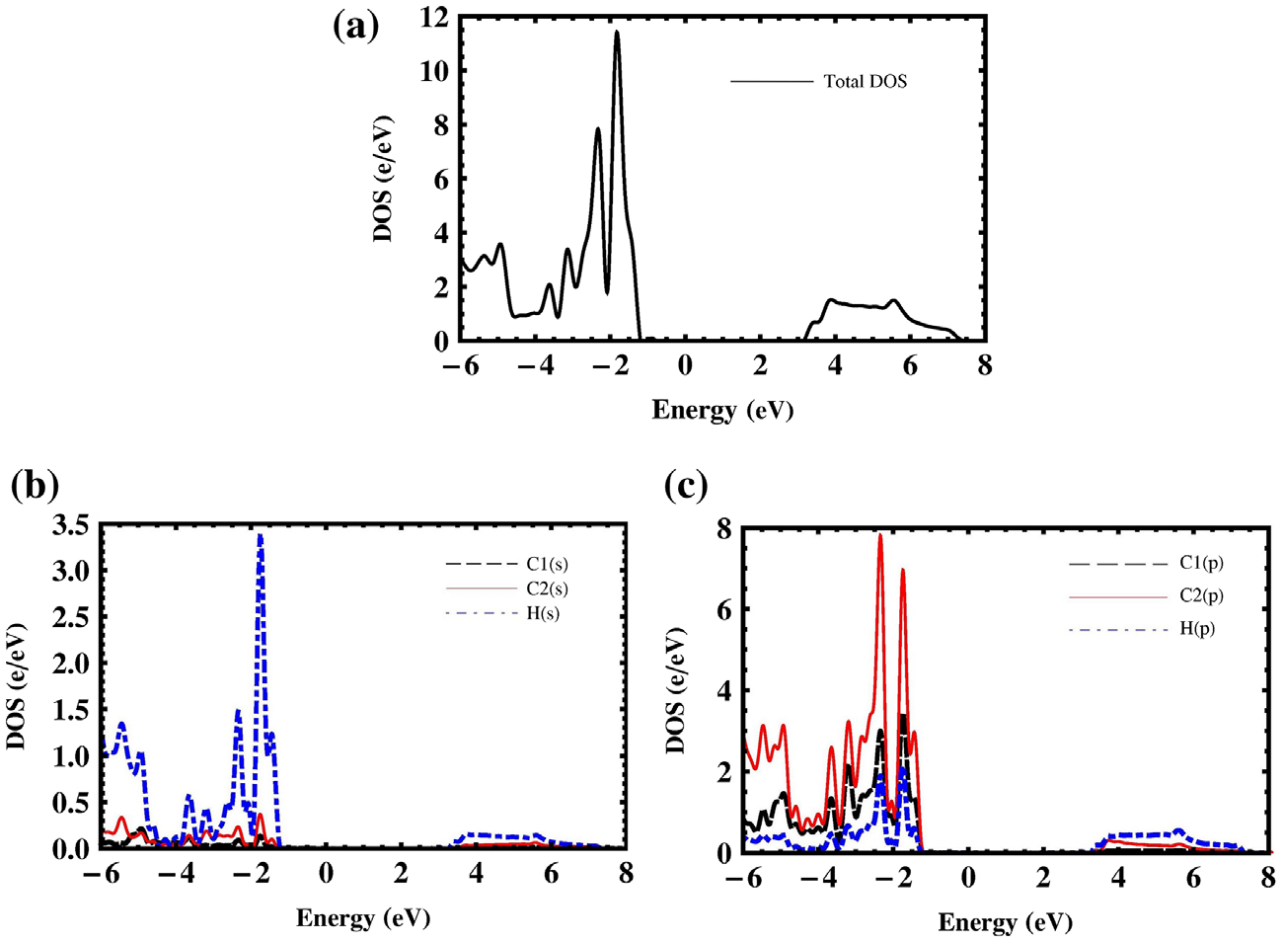
(a) Total density of states, (b) partial density of s-states and (c) p-states of penta-graphane, (black, blue and red corresponds to C1, C2 and hydrogen atoms, respectively).

The differences between the minimum and the maximum of the highest valence band in penta-graphane and penta-graphene are 0.37 and 0.58 eV, respectively, which lead to a higher total density of states (DOS) near Fermi level for penta-graphane (Figure 4(a)) in comparison with penta-graphene. As is stated in [34], there are some initial requirements for high superconducting transition temperature: *σ* electrons at the Fermi surface, large bond-stretching phonon frequencies and large electron DOS at the Fermi energy. In penta-graphane, we have sp^3^ bonds (Figure 1); therefore, there are σ electrons at the Fermi surface. We see that penta-graphane has large bond-stretching phonon frequency (cf. Figure 6(a)). Here, if we lower Fermi-energy, we will have strong DOS at the Fermi level; therefore, from these properties, we could expect that a decrease in the Fermi energy may increase the superconducting transition temperature, similar to graphane.[35] Decreasing Fermi energy could be obtained, similar to graphane,[34,36] by p-doping or hole-doping of the penta-graphane. For instance, superconducting in hole-doped graphane for the doping range 1–8% was studied in [36], which showed that the electron–phonon interaction in hole-doped graphane is much stronger than that in graphane, due to a strong sp^3^ bonding. Also, the obtained results suggest that hole-doped graphane could potentially be a superconductor with a higher transition temperature. The analysis of partial DOS indicates that the total DOS near VBM and CBM are mostly related to S and P states, and the total DOS below Fermi level is mainly composed of H(s) and C2(p) (Figure 4(b) and (c)).

**Figure 5.**
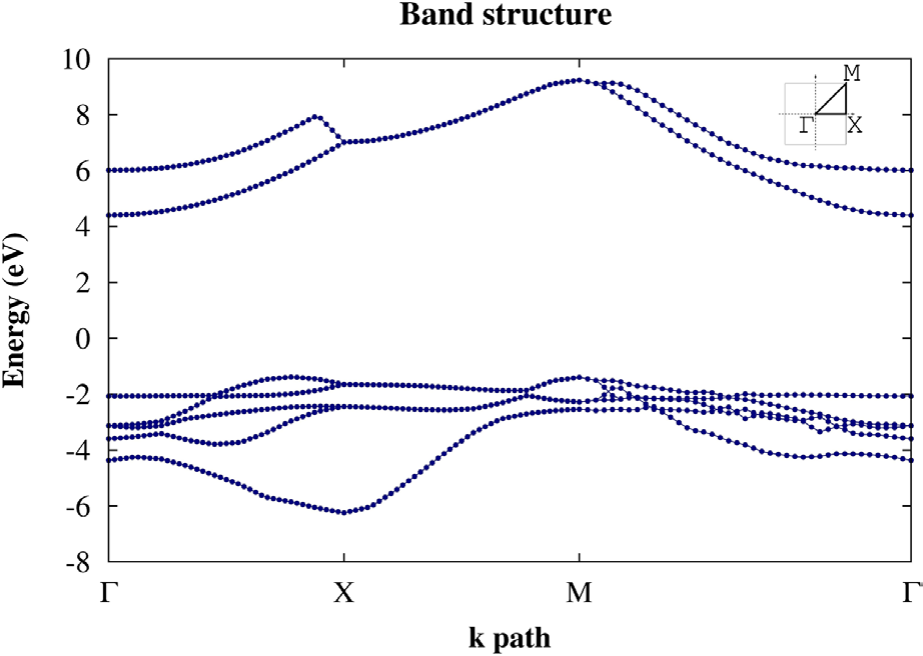
The band structure of penta-graphane calculated within the G_0_W_0_ approximation.

For a better approximation, we use G_0_W_0_. The G_0_W_0_ corrections are obtained using 18 × 18 × 1 *k* points in Brillouin zone and 20 Hartree cutoff energy. The computations converge at *n*=50 bands for computing W and self-energy. Using interpolation, we have plotted the band structure of penta-graphane in Figure 5. This plot shows that penta-graphane has an indirect band gap and the band gap of penta-graphane in G_0_W_0_-GGA is 5.78 eV. The G_0_W_0_ leads to a lower energy for the valence band and a higher energy for the conduction band. Therefore, the G_0_W_0_ band gap is bigger than DFT. The G_0_W_0_ and DFT valence bands have similar dispersion and the curvature of the valence band does not change in Figure 5; therefore, the hole effective mass in k_x_ direction is the same (0.58m_0_ ^1^) for DFT and G_0_W_0_. Hole effective mass in k_y_ direction is 2.21 m_0_ for G_0_W_0_, and 4.62 m_0_ for DFT. Electron effective mass in k_x_ (k_y_) direction is 0.9 m_0_ (0.65 m_0_) for G_0_W_0_ and 1.4 m_0_ (1.2 m_0_) for DFT.

**Figure 6.**
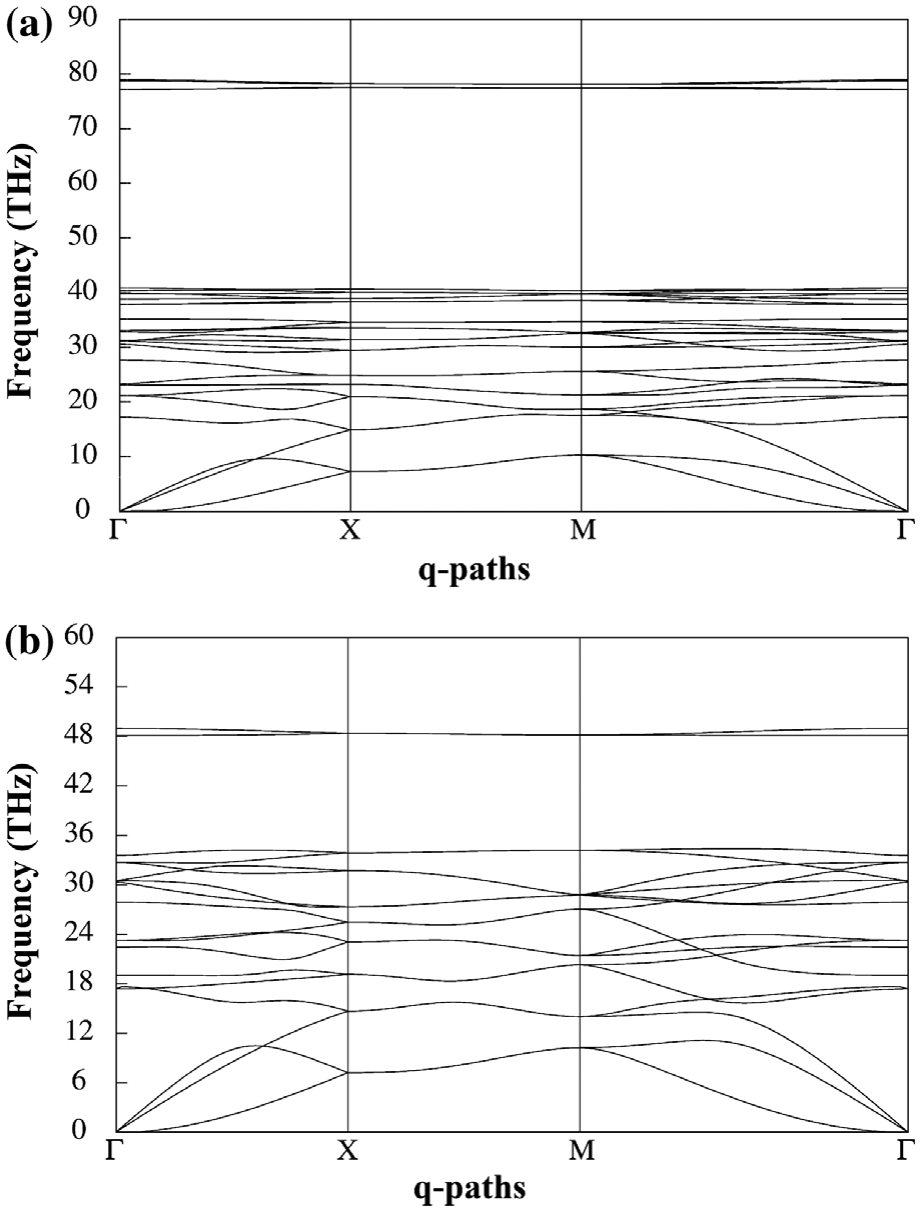
(a) Phonon dispersion of penta-graphane and (b) penta-graphene.

Note that the band gap of penta-graphene in G_0_W_0_-GGA is 4.14 eV.[16] The reason for this band gap opening, when penta-graphene is hydrogenated and forms penta-graphane, is that sp^3^ bonds are saturated and we have not any free *π* orbital. The fluctuation of valence band is due to the perturbation solving of G_0_W_0_, which occurs when bands are very close together. Comparing the band gap of carbon allotropes, penta-graphane has the largest band gap, a bit larger than diamond and graphane G_0_W_0_ (5.56 eV and 5.66 eV, respectively [37,38]). HOMO (highest occupied molecular orbital) energy is –1.38 eV and LUMO (lowest unoccupied molecular orbital) energy is 4.39 eV. Fermi energy is in the band gap and the band gap (5.78 eV) is difference between HOMO and LOMO energies. In penta-graphane carbon atoms are sp^3^-hybridized and electron bonds are saturated with hydrogen atoms. Therefore, penta-graphane does not have any free electrons and is a good band insulator with band gap 5.78 eV. From Figure 5, the G_0_W_0_ (GGA-RPBE) optical gap of penta-graphane is 6.31 eV (196.48 nm), which is placed in a farther ultraviolet region wavelength than penta-graphene.

### 3.2. Phonon structure

We have calculated the phonon dispersion of penta-graphane with the ABINIT code as shown in Figure 6. The energy cutoff is 50 Hartree. The vacuum between layers is 10 Å (the same as the electronic calculations). The dynamical properties are calculated with the density-functional perturbation theory.[26] We use Fourier based interpolation of the interatomic force constants obtained on a 9 × 9 × 1 grid of q-points.

The 10 atoms per primitive unit cell give 30 vibration modes which have three acoustic modes with zero frequency at Γ point, and 27 optic modes. The absence of any imaginary frequency signifies that the penta-graphane is structurally stable. The high frequency modes are higher in frequency in comparison with penta-graphene,[15] and these high frequency modes show that penta-graphane is a covalent solid.

**Figure 7.**
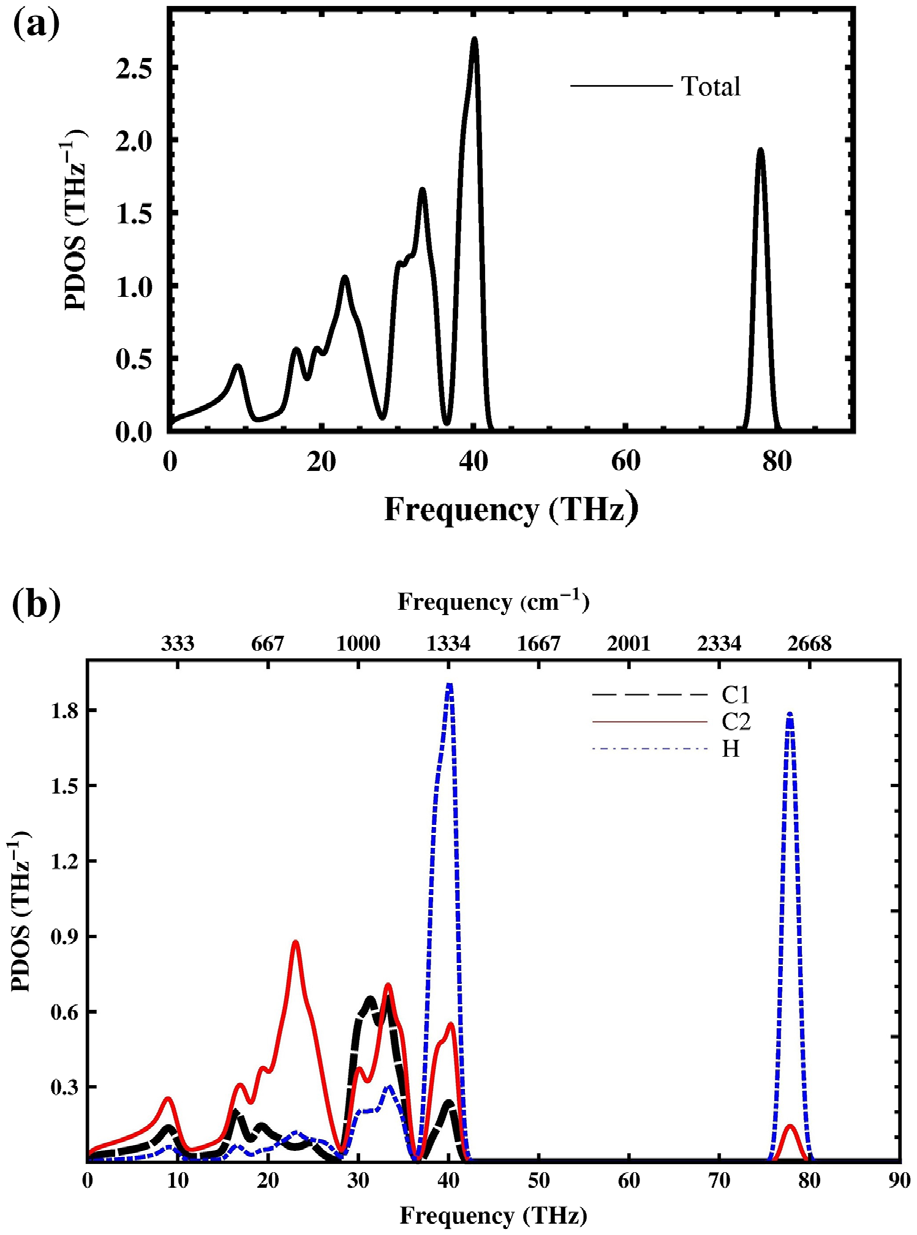
(a) Total phonon density of states and (b) partial contribution from C1, C2 and H (PDOS) of penta-graphane.

The total phonon density of states (PDOS) and partial PDOS are shown in Figure 7. The low frequency bands (acoustics frequency) (0–18 THz) correspond to C1 and C2 vibrations. Hydrogen has lighter mass, so the high frequency bands are mainly H-like. The frequency bands between 18 and 28 THz are C2-like and in the frequency bands between (28–38 THz) C1 and C2 have nearly the same vibrations. The analysis of partial density of states shows that penta-graphane has C-H (sp^3^) stretching modes around 78 THz (2600 cm^−1^).

From the PDOS we can calculate the specific heat capacity at constant volume by taking the first derivation of the total energy:

(4) $$C_{V}\left(T\right)=k_{B}\int_{0}^{\omega_{max}}{\left(\frac{\hbar \omega }{k_{B}T}\right)}^{2}\frac{\text{exp}\left(\frac{\hbar \omega }{k_{B}T}\right)}{{\left(\exp\left(\frac{\hbar \omega }{k_{B}T}\right)-1\right)}^{2}}\;g\left(\omega \right)\text{d}\omega$$

**Figure 8.**
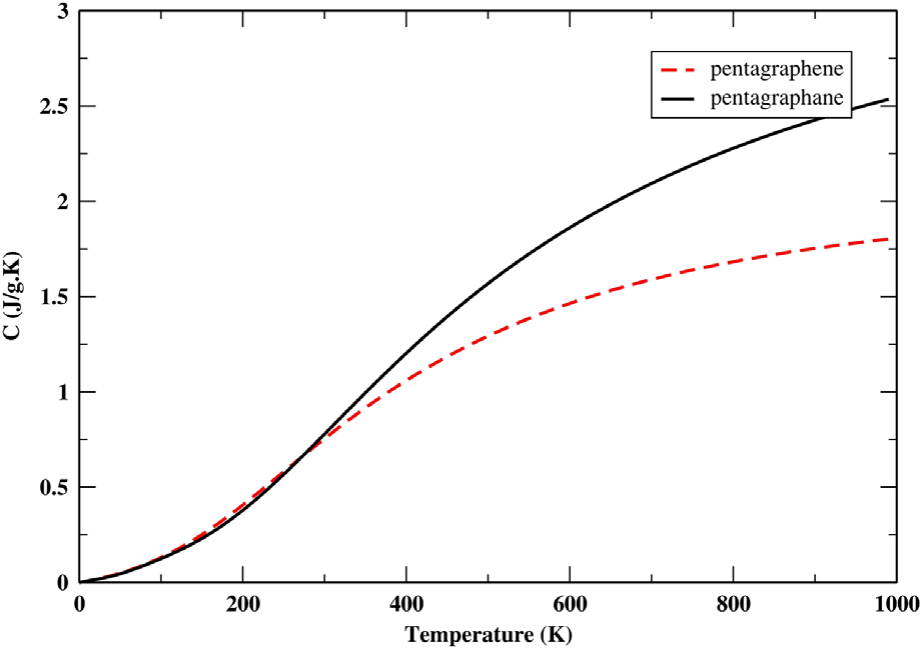
The specific heat capacity at constant volume of penta-graphene and penta-graphane.

We compare the specific heat capacity at constant volume of penta-graphene and penta-graphane in Figure 8.

The reason for the higher specific heat capacity in comparison with penta-graphene is the existence of hydrogen bonds.

The specific heat capacity consists of phonon and electron contributions. The electron specific heat capacity is proportional to the density of states at Fermi level. Therefore the specific heat capacity of penta-graphane is dominated by the phonon contribution at all temperatures in penta-graphane.

We could call penta-graphane a ‘graphane allotrope’. Penta-graphane and graphane have only sp^3^ bonds. The specific heat capacity diagrams of penta-graphane and graphane are very similar.[39] In addition, although both penta-graphane and graphane have large bond-stretching phonon frequencies, due to the dispersionless valence band near the Fermi level, penta-graphane has higher total density of states at the valence band near Fermi level in comparison to graphane. Therefore, we could expect that penta-graphane has a high superconducting transition temperature, similar to graphane.[34,36]

## 4. Conclusions

In this paper we have considered a form of hydrogenated penta-graphene, penta-graphane. This structure is more stable than the corresponding hydrogenated graphane and we could expect that hydrogenation of graphene, by satisfying some experimental requirements, may lead to penta-graphane. The band structure of penta-graphane has been computed by DFT and G_0_W_0_ which we have shown in Figures 3 and 5, respectively. We have found that penta-graphane is a good insulator with 5.78 eV band gap. Penta-graphane has diamond-like structure with sp^3^ hybridization. It has high total density of states near Fermi level, therefore, lowering Fermi energy leads to strongly total DOS at the Fermi level in penta-graphane. The phonon dispersion of penta-graphane has been shown in Figure 6(a). The absence of any imaginary frequency signifies the structural stability of the penta-graphane. Penta-graphane has a high specific heat capacity and hence can be used for storing and transferring energy. Since penta-graphane has thermodynamic, mechanical and structural stability, we can expect interesting applications of this novel structure.

## Disclosure statement

No potential conflict of interest was reported by the authors.

## Supplemental material

The supplemental material for this paper is available online at http://dx.doi.org/10.1080/14686996.2016.1219970.

## Supplementary Material

Supplementary_Information.docx

## Footnotes

^1^ m_0_=$9.11\times 10^{-31}$ kg (free electron mass)
