# Supplementary material for: Studying the electronic and phononic structure of penta-graphane
Source: Sci Technol Adv Mater. 2016 Oct 7;17(1):610–7. doi: 10.1080/14686996.2016.1219970 (PMC5102001; doi:10.1080/14686996.2016.1219970)
Supplement: Supplementary_Information.docx [file tsta_a_1219970_sm6246.docx]

Supplementary Information

**Studying the electronic and phononic structure of penta-graphane**

Hamideh Einollahzadeh^1^, Seyed Mahdi^2^ Fazeli, Reza S. Dariani^1^

^1^Department of Physics, Alzahra University, Tehran, 1993893973, Iran

^2^Department of Physics, University of Qom, Qom, 3716146611, Iran

**Additional data**

Table S1. Crystal structure and binding energy of penta-graphane

| **parameter** | **Penta-graphane** |
| --- | --- |
| a(Å) | 3.60 |
| b(Å) | 3.60 |
| c(Å) | 10 |
| $\alpha\left( \text{degrees} \right)$ | 90 |
| $\beta\left( \text{degrees} \right)$ | 90 |
| $\gamma\left( \text{degrees} \right)$ | 90 |
| Cell volume | 129.6 |
| Total energy(eV/unit cell) | -1053.85 |
| Binding energy(eV/ unit cell) | -67.3 |

Table S2. Atomic coordinates of penta-graphane in angstrom

|  | x | y | z |
| --- | --- | --- | --- |
| C1 | -0.042 | -0.042 | 0.270 |
| C2 | 1.756 | 1.756 | 0.269 |
| C3 | 1.215 | 0.498 | -0.454 |
| C4 | 2.297 | 3.014 | -0.454 |
| C5 | 3.014 | 1.215 | 0.994 |
| C6 | 0.498 | 2.297 | 0.994 |
| H1 | 0.990 | 0.724 | -1.538 |
| H2 | 2.522 | 2.789 | -1.538 |
| H3 | 2.789 | 0.990 | 2.078 |
| H4 | 0.724 | 2.522 | 2.078 |

Table S3. Crystal structure and binding energy of C_6_H_4_  (not fully hydrogenated graphane)

| **parameter** | _C6H4 (not fully hydrogenated graphane)_ |
| --- | --- |
| a(Å) | 4.22 |
| b(Å) | 4.22 |
| c(Å) | 10 |
| $\alpha\left( \text{degrees} \right)$ | 90 |
| $\beta\left( \text{degrees} \right)$ | 90 |
| $\gamma\left( \text{degrees} \right)$ | 120 |
| Cell volume | 152.3 |
| Total energy(eV/unit cell) | -1052.30 |
| Binding energy(eV/ unit cell) | -65.7 |

Table S4. Atomic coordinates of C_6_H_4_(not fully hydrogenated graphane) in angstrom

|  | x | y | z |
| --- | --- | --- | --- |
| C1 | 1.996 | 1.204 | -0.348 |
| C2 | 4.232 | 2.441 | 0.381 |
| C3 | 4.232 | -0.032 | 0.381 |
| C4 | 2.806 | 2.441 | -0.381 |
| C5 | 2.806 | -0.032 | -0.381 |
| C6 | 5.042 | 1.204 | 0.348 |
| H1 | 3.064 | 2.757 | -1.407 |
| H2 | 3.064 | -0.348 | -1.407 |
| H3 | 3.974 | 2.757 | 1.407 |
| H4 | 3.974 | -0.349 | 1.407 |

Table S5. Elastic constants for penta-graphane (unit:GP.nm) (at fixed electric field boundary condition):

|  | C_11_=C_22_ | C_12_ | C_13_ | C_33_ | C_44_ | C_55_ | C_66_ | Poisson's ratio | in-plane Young's modulus |
| --- | --- | --- | --- | --- | --- | --- | --- | --- | --- |
| relaxed ion | 223.0 | 54.8 | -0.4 | -0.1 | -0.5 | -0.4 | 156.9 | 0.24 | 209 |
| clamped ion[1] | 303.0 | 46.5 | 39.0 | 279.9 | 138.1 | 138.1 | 176.9 | 0.15 | 295 |

The in-plane Young’s modulus: $\text{E = (}\text{C}_{\text{11}}^{\text{2}}\text{-}\text{C}_{\text{12}}^{\text{2}}\text{)/}\text{C}_{\text{11}}$

Table S6. Comparison the energy band in DFT and G_0_W_0_ approximation at gamma point in penta-graphane (all in [eV]):

| $\Gamma$= (0.000, 0.000, 0.000) | | | |
| --- | --- | --- | --- |
| Band | E_DFT_ | $\text{E}_{\text{G}_{\text{0}}\text{w}_{\text{0}}}$ | $\text{E}_{\text{G}_{\text{0}}\text{w}_{\text{0}}}$-E_DFT_ |
| 10 | -3.620 | -3.874 | -0.254 |
| 11 | -3.537 | -4.079 | -0.542 |
| 12 | -3.054 | -3.276 | -0.222 |
| 13 | -3.015 | -3.233 | -0.218 |
| 14 | -1.747 | -1.946 | -0.198 |
| 15 | 3.253 | 4.362 | 1.108 |
| 16 | 3.583 | 6.061 | 2.479 |
| E_DFT__gap | | 5.001 | |
| $\text{E}_{\text{G}_{\text{0}}\text{w}_{\text{0}}}$_gap | | 6.307 | |
| $\Delta$E_gap_ | | 1.307 | |

Fig. S1. Internal energy E of penta-graphane


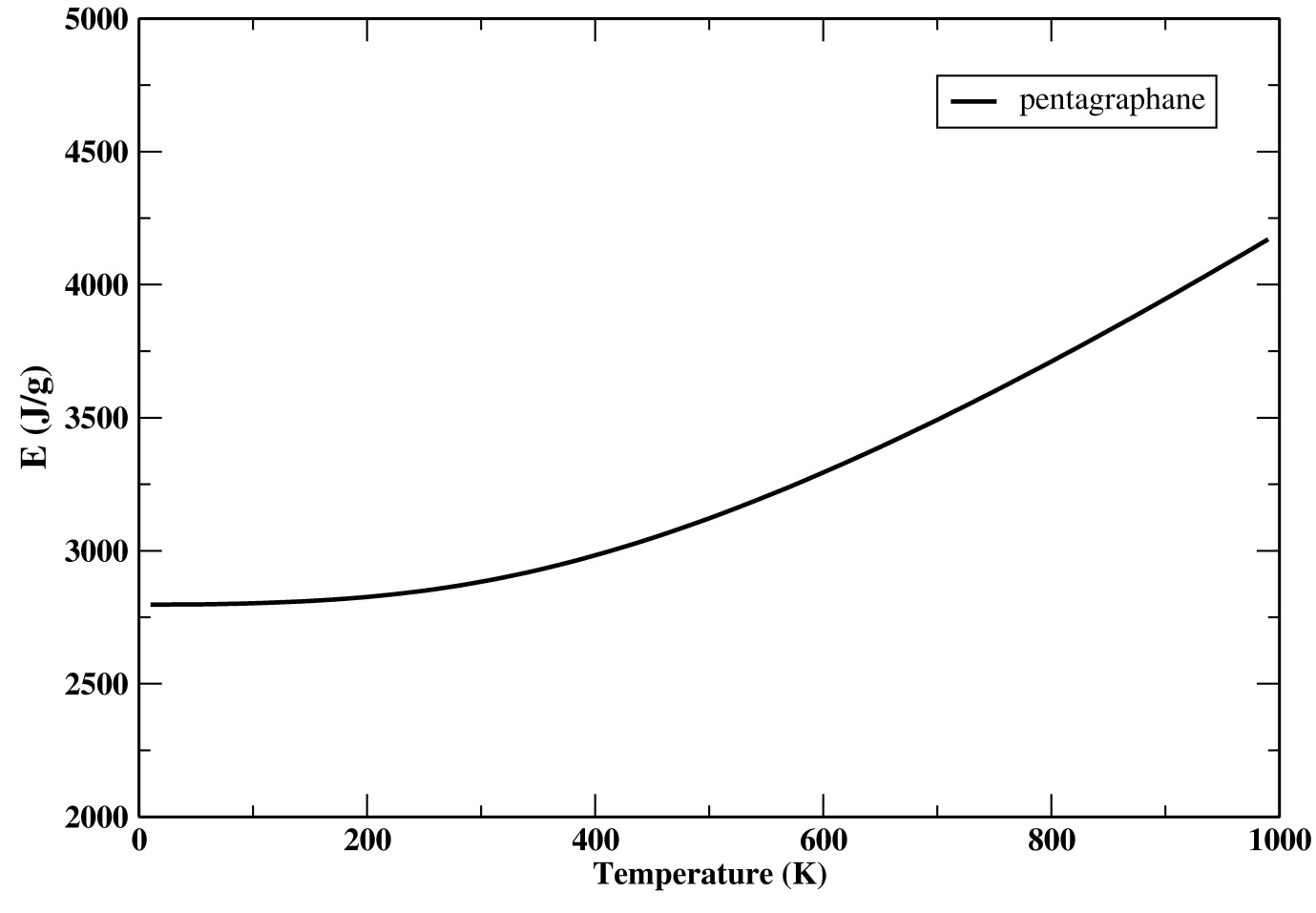


The phonon contribution to the internal energy [2] :

$$\Delta E=3nN\frac{\hbar}{2}\int_{0}^{\omega_{L}} \omega\coth(\frac{\hbar\omega}{2k_{B}T}) g\left( \omega\right)d\omega$$

**References**

[1] C. Tholander, “Piezoelectricity, Phase Stability, and Surface Diffusion in Multicomponent Nitrides”, Linköping University Electronic Press (2016)

[2] C. Lee and X. Gonze, Physical Review B 51(13) (1995) 8610
